# Impact of small vessel disease in the brain on gait and balance

**DOI:** 10.1038/srep41637

**Published:** 2017-01-30

**Authors:** Daniela Pinter, Stuart J. Ritchie, Fergus Doubal, Thomas Gattringer, Zoe Morris, Mark E. Bastin, Maria del C. Valdés Hernández, Natalie A. Royle, Janie Corley, Susana Muñoz Maniega, Alison Pattie, David A. Dickie, Julie Staals, Alan J. Gow, John M. Starr, Ian J. Deary, Christian Enzinger, Franz Fazekas, Joanna Wardlaw

**Affiliations:** 1 Department of Neurology, Medical University of Graz, Graz, 8036, Austria; 2 Centre for Cognitive Ageing and Cognitive Epidemiology, University of Edinburgh, Edinburgh, EH8 9YL, UK; 3 Department of Psychology, University of Edinburgh, Edinburgh, EH8 9YL, UK; 4 Brain Research Imaging Centre, University of Edinburgh, Edinburgh, EH4 2XU, UK; 5 Centre for Clinical Brain Sciences, University of Edinburgh, Edinburgh, EH4 2XU, UK; 6 Department of Neurology, Maastricht University Medical Centre, Maastricht, 6211, Netherlands; 7 Department of Psychology, Heriot-Watt University, Edinburgh, EH14 4AS, UK; 8 Alzheimer Scotland Dementia Research Centre, Department of Psychology, University of Edinburgh, Edinburgh, EH8 9YL, UK; 9 Division of Neuroradiology, Vascular and Interventional Neuroradiology, Department of Radiology, Medical University of Graz, Graz, 8036, Austria

## Abstract

Gait and balance impairment is highly prevalent in older people. We aimed to assess whether and how single markers of small vessel disease (SVD) or a combination thereof explain gait and balance function in the elderly. We analysed 678 community-dwelling healthy subjects from the Lothian Birth Cohort 1936 at the age of 71–74 years who had undergone comprehensive risk factor assessment, gait and balance assessment as well as brain MRI. We investigated the impact of individual SVD markers (white matter hyperintensity – WMH, microbleeds, lacunes, enlarged perivascular spaces, brain atrophy) as seen on structural brain MRI and of a global SVD score on the patients’ performance. A regression model revealed that age, sex, and hypertension significantly explained gait speed. Among SVD markers white matter hyperintensity (WMH) score or volume were additional significant and independent predictors of gait speed in the regression model. A similar association was seen with the global SVD score. Our study confirms a negative impact of SVD-related morphologic brain changes on gait speed in addition to age, sex and hypertension independent from brain atrophy. The presence of WMH seems to be the major driving force for SVD on gait impairment in healthy elderly subjects.

Gait and balance impairment is highly prevalent in older people, restricts independence and increases the risk of falls, institutionalization and mortality^1^. Impairment increases rapidly in prevalence from around 15% at the age of 60 to >50% in individuals aged 80 years and older^2^,^3^,^4^. Besides increasing age, there are multiple contributing factors causing gait and balance disturbances, including vascular risk factors (e.g. high blood pressure or diabetes)^5^,^6^. Occurrence of cerebrovascular disease in the elderly, particularly of cerebral small vessel disease (SVD), also appears to play some role in the development of gait and balance impairment^7^,^8^,^9^. Cerebral SVD refers to a group of pathological processes with various aetiologies^7^ which appear on magnetic resonance imaging (MRI) as white matter hyperintensities (WMH), microbleeds, lacunes of presumed vascular origin and enlarged perivascular spaces (PVS)^7^,^10^,^11^,^12^. SVD has also been suggested to cause brain volume loss^13^,^14^, but defined cut-offs regarding whether atrophy is related to “normal” aging or SVD are missing. Nevertheless, total brain volume could act as an important mediator in the relation between SVD and gait or balance function^15^ and needs to be considered when examining the impact of SVD features on gait and balance function.

Despite being the second most common problem after impaired cognitive functioning^16^, gait and balance disturbances have rarely been a target outcome variable in large population-based studies examining the clinical consequences of SVD^17^. Moreover most investigations have focused only on the association between gait and balance impairment with single MRI markers of SVD^15^,^18^,^19^,^20^,^21^,^22^. Hence, it is unclear whether the strength of the association with disturbed gait and balance varies between individual features of SVD as they reflect distinct pathologies with a possibly different impact on brain function. Alternatively, and if they exerted additive damaging effects, a simple sum score of SVD markers could capture the burden from cerebral changes induced by SVD in a more global manner and provide more robust associations as recently shown for the relation between SVD and cognitive impairment^23^,^24^.

With these considerations we aimed to (a) demonstrate the potential link between distinct SVD MRI markers and gait or balance function in healthy subjects. (b) Furthermore, we explored the differential link between single versus combined SVD markers on gait and balance function. Due to the fact, that multiple variables could lead to gait and balance impairment in healthy elderly subjects, we additionally examined (c) the impact of single versus combined SVD markers beyond demographics, risk factors and atrophy. As different parameters (e.g. a simple visual rating scale vs. volumetric assessment)^25^ might be used to describe WMH burden, we included both visual rating and volumetric data in our analyses. To reduce confounding effects, we investigated these questions in a cohort of independent community-dwelling healthy older subjects with the same year of birth.

## Methods

### Participants

Data of 866 community-dwelling healthy subjects from the Lothian Birth Cohort 1936 (LBC1936) were available for this investigation. More detailed information of the recruitment and study procedure can be found in Deary *et al*.^26^,^27^. 680 (79%) of those individuals had undergone comprehensive risk factor assessment, gait and balance assessment, as well as brain MRI and thus could be used for our analyses. The main loss of subjects was in those who opted not to have a brain MRI or if the MRI data was not suitable. Two further participants were excluded because of a diagnosis of Parkinson’s disease, leaving a final sample of 678 subjects. Assessments used for the present study were performed at a visit at about age 73 (age range = 70.96 to 74.21 years).

### Standard protocol approvals, registrations, and patient consents

Ethics permission for the study protocol was obtained from the Multi-Centre Research Ethics Committee for Scotland (MREC/01/0/56) and from Lothian Research Ethics Committee (LREC/2003/2/29). Experimental protocols were approved by the Multi-Centre Research Ethics Committee for Scotland (MREC/01/0/56) and Lothian Research Ethics Committee (LREC/2003/2/29). The research was carried out in compliance with the Helsinki Declaration. All experiments were performed in accordance with relevant guidelines and regulations. All participants gave written, informed consent.

### General assessment

An extensive description of all variables obtained for the LBC1936 study can be found in Deary *et al*.^26^ for clinical and Wardlaw *et al*.^28^ for imaging variables. All participants underwent a medical interview and physical examination. Disease history and vascular risk factors (e.g. smoking, high blood pressure, diagnosis of diabetes, high cholesterol) were obtained in a structured interview.

### Assessment of gait and balance function

Gait speed was assessed by the six-meter walk test, a common assessment used in research studies to access physical function. Furthermore, two subtests (chair-stands and standing balance) of the Short Physical Performance Battery were applied. Please see Supplementary Information for more details.

### Brain MRI acquisition

The brain imaging protocol for the study has been described previously^28^. All participants were scanned on a General Electric 1.5 T clinical MRI scanner (Signa Horizon HDx) operating in research mode. For this study, we used axial T2, T2*, fluid-attenuated inversion recovery (FLAIR) and T1-weighted sequences.

### MRI rating and composition of SVD score

MRI images were rated for the presence of WMH, perivascular spaces (PVS), microbleeds and lacunes using a standardized protocol, with validated visual scales and these ratings also served to create a global SVD scale ranging from 0–4 as described previously^12^,^24^,^29^,^30^,^31^. In short, deep WMH and periventricular hyperintensities (PVH) were graded on the Fazekas scale (each 0–3) using FLAIR- and T2-weighted sequences and one point was awarded on the SVD scale when early confluent to confluent deep WMH (WMH score 2 or 3) and/or irregular PVH extending into the deep white matter (PVH score 3) were present. PVS defined as small (<3 mm) punctate hyperintensities on T2-weighted images were rated from 0 (absent) to 4 (severe) and one point on the SVD scale was awarded when moderate to severe (grade 2–4) PVS in the basal ganglia were present. Microbleeds (CMB) were defined as small (<10 mm), homogeneous, round foci of low signal intensity on T2*-weighted images and the presence of one or more CMB gave one point on the SVD scale. Lacunes were defined as small (<20 mm in diameter), subcortical lesions of similar signal to CSF and ≥1 lacune was one point on the SVD scale. We also assessed the WMH volume in cm^3 ^32 and calculated the brain volume normalized by intracranial volume^28^,^33^.

### Statistical Analysis: with gait

We used the Statistical Package of Social Science (IBM SPSS Statistics 23, SPSS Inc., Chicago, Illinois, USA) for T-Tests, nonparametric analysis (e.g. Mann-Whitney U for standing balance) and correlation analysis. First we examined the associations between demographic and vascular risk factor variables (e.g. sex, age, smoking), atrophy (NBV) and SVD markers with the dependent variables (gait speed, chair-stands test or standing balance) using Pearson correlation, Spearman correlation or point-biserial correlation. To investigate the association of the individual SVD markers and of the global SVD score or of WMH volume withgait speed and chair-stands, we applied hierarchical linear regression analysis. We controlled for sex and age in the first step of our hierarchical regression analysis, and vascular risk factor variables in the second step, adding MRI-parameters in the third step. A negative binomial regression analysis was used for prediction of standing balance function. The level of significance was set at 0.05. Bonferroni-adjusted level of significance is indicated were applicable.

## Results

### Sample characteristics

Table 1 gives information on the demographics, risk factor variables and gait and balance function of the study sample. Table 2 provides a more detailed overview regarding the prevalence of single SVD markers and an overview on the MRI-characteristics of investigated individuals including the contribution of the individual MRI markers to the global SVD score. Overall, most of the subjects had a SVD score of 0 or 1 (80.8%), while scores 2–4 were less frequent (19.2%).

### Univariate correlations of gait speed, chair-stands and balance test with demographics, risk factors and SVD related abnormalities

Regarding demographics and risk factors, correlational analysis showed that gait speed was significantly slower in older subjects, females, subjects with high blood pressure (HBP) or diabetes (see Table S1 in the Supplementary Information). Furthermore, performance on the chair-stands test was significantly worse in older subjects, smokers and subjects with diabetes. Standing balance was significantly worse in female subjects, subjects with HBP or diabetes.

Regarding focal SVD related abnormalities and normalized brain volume (NBV), correlational analysis showed that gait speed was significantly slower in subjects with higher WMH and PVS scores, higher WMH volume, lower NBV and a higher global SVD score (see Table S2 in the Supplementary Information). Furthermore, performance on the chair-stands test was worse in subjects with higher WMH score and a higher global SVD score. Performance on the standing balance test was worse in subjects with higher WMH volume and a higher global SVD score.

### Association of SVD related abnormalities with gait speed, chair-stands and balance test

Using a linear regression analysis model to assess the contribution of individual SVD markers to patients’ performance only the WMH score (*R*^*2*^ = 1.3%) and WMH volume (*R*^*2*^ = 2.3%) were associated significantly with gait speed following Bonferroni-adjustment (Table 3). None of the other SVD markers (microbleeds, lacunes of presumed vascular origin, PVS) significantly predicted gait. Gait speed was also significantly and separately predicted by the global SVD score (*R*^*2*^ = 1.4%). Performance on the chair-stands test was predicted by the global SVD score (*R*^*2*^ = 0.6%) and the WMH score (*R^2^* = 0.7%) but significance did not withstand Bonferroni-adjustment. The negative binomial regression model revealed no significant prediction of performance on the standing balance test from any SVD marker (Table 3).

### Association of SVD related abnormalities with gait speed and balance function in addition to demographics, risk factors and atrophy

We used a hierarchical linear regression model to assess whether SVD markers independently predict gait speed, chair-stands and balance function in addition to sex, age, risk factors and NBV. The extended regression model revealed that age (age (*p* < 0.001; older subjects performed worse) and sex (*p* < 0.001; women performed worse; *R*^*2*^ = 6.8%), as well as HBP (*p* = 0.003) significantly contributed to prediction of gait speed. Standardized beta-values (*βj*) and adjusted R^2^ (variance accounted for) are shown in Table 4. Incrementally, MRI-parameters added to the explanation. NBV and single SVD markers (WMH score or WMH volume) independently explained gait. The hierarchical regression model showed that in addition to NBV (*p* = 0.010), the WMH score (*p* = 0.010; ∆*R*^*2*^ = 1.5%) and WMH volume (*p* = 0.001; ∆*R*^*2*^ = 2.9%) independently added to prediction of gait speed. Similar scores were observed if single WMH markers were replaced by the global SVD score in the regression model (*p* = 0.003; ∆*R*^*2*^ = 2.0%).

For the chair-stands test, none of the single SVD markers, be it single or combined, explained incremental variance after controlling for demographics (sex: *p* < 0.001, women performed worse; *R*^*2*^ = 0.1%), risk factors and NBV (Table 4). None of the demographic variables, risk factors and MRI parameters significantly predicted performance on the standing balance test.

### Sensitivity analysis for subjects with moderate to severe SVD scores

To allow some comparison with previous cohort studies (e.g. LADIS, RunDMC)^18^,^19^,^20^, we also performed additional analysis including only subjects with moderate to severe (≥2) global SVD scores (N = 127). In this subsample demographics and risk factors did not significantly predict gait speed, whereas NBV (6%) and the global SVD score (6.4%) were significant in accounting for 12.4% of the variance (*βj* = 0.22, *p* = 0.013). WMH score (*p* = 0.216) and volume (*p* = 0.309) did not significantly explain gait speed in subjects with moderate to severe SVD. No variable significantly explained performance on the chair-stands test or standing balance test.

## Discussion

This study confirms that SVD-related brain changes are associated with worse performance in gait speed, the chair-stands test and standing balance in older independent community-dwelling subjects. Regression models showed that a single marker of SVD (i.e. the WMH score or WMH volume) and the global SVD score consistently explained gait speed (*R*^*2*^ = 1.3–2.3%). Results for the chair-stands test pointed in the same direction: The WMH score and the global SVD score were related to worse performance (*R*^*2*^ = 0.6–0.7%), but these results did not withstand Bonferroni-adjustment. No significant explanation of standing balance for any SVD marker (single or combined) was observed.

As expected demographics (e.g. sex, age) and risk factors (high blood pressure) had an impact on gait speed in healthy older subjects, but SVD related abnormalities still additionally explained changes in gait independently from atrophy. Overall, compared to previous studies the contribution to explain changes in gait speed appears rather low. There are two likely explanations for this finding:

First, in comparison to previous cohort studies (e.g. LADIS, RunDMC)^18^,^20^ which partly included neurologic ‘patients’, our community-dwelling, non-disabled older subjects had relatively low SVD burden. 44.4% had a global SVD score of ‘zero’ indicating only minor individual SVD markers. While this certainly reduces the strengths of correlations it is thus reassuring that we could still confirm earlier findings of a correlation between SVD and gait and balance disorders in a group of individuals without possible confounders such as other neurologic disorders and age. However, as explanation of variance was comparable between the WMH score and the global SVD score, it seems that WMH is the major driving force to impact gait function in a sample of healthy community-dwelling subjects^18^,^19^,^34^. This likely comes from the associated rather diffuse and widespread effects of such abnormalities^17^,^35^ while other SVD features (e.g. lacunes or microbleeds) may have been not severe enough to cause a functional impact on their own.

To allow for further appreciation of the impact of SVD severity and comparison with the above mentioned studies, we additionally examined the relationship between gait and balance when considering only subjects with moderate to severe SVD scores (global SVD score >=2). Prediction improved substantially for gait speed, showing that the global SVD score accounted for 6.4% of the variance in addition to NBV (6%), whereas demographics and risk factors did not add to the explanation. Furthermore, in this more severely impaired cohort the impact WMH (score or volume) did not reach significance, suggesting the occurrence of additive damaging effects of multiple single SVD features on gait function and it is probably in this setting where a global SVD score may be of value.

Opposed to patient cohorts, other variables such as physical fitness, visual acuity, chronic pain or cognitive function might stronger influence gait and balance function. However, as the main focus of our study was examining the relationship between single compared to combined SVD markers with and gait or balance function, we focused on the major variables influencing gait and balance function (e.g. age, sex, vascular risk factors) in our study. Secondly, the clinical scores used to assess balance function seem to be insensitive to capture SVD related balance impairments, which are likely to be rather subtle in healthy community-dwelling subjects, presenting earlier stages of SVD^36^. Compared to prediction of gait speed, results for the chair-stands test and standing balance test were less conclusive. Performance on the chair-stands test might be stronger dependent on physical factors (e.g. fitness, muscle, lung function)^37^. Also, a more detailed assessment of standing balance (such as dynamic posturography)^38^ might be needed. The ordinal scale of the standing balance test might not be sensitive enough to assess balance impairment in our sample and more informative for clinical cohorts.

Surprisingly, despite the narrow range (71–74 years), age was still a significant contributor to the variance explained in gait function. Hence, controlling for age effects seems to be crucial in advanced age, even in a very narrow age-range birth cohort^17^. Also a previous study^4^ showed that gait disorders are prevalent in about 11% of 60–69 year old subjects, rising to 38% in 70–79 year old subjects, suggesting a steep increase of gait impairment from one decade to another. Furthermore and in line with previous studies, high blood pressure^6^ predicted worse performance in gait function.

Although studies have reported associations between atrophy and SVD^13^,^14^, specifications and defined cut-offs regarding whether atrophy is related to “normal” aging or SVD are missing. Therefore, in this study, we focused on the focal SVD markers only. Nevertheless, we included total brain volume in our hierarchical regression analysis and found that SVD-related brain changes and brain volume are both significantly, but independently explain gait function.

In conclusion our study confirms a negative impact of SVD-related morphologic brain changes on gait speed in addition to age, sex and hypertension and independent from normalized brain volume. The presence of WMH seems to be the major driving force for gait impairment in healthy elderly subjects.

## Additional Information

**How to cite this article**: Pinter, D. *et al*. Impact of small vessel disease in the brain on gait and balance. *Sci. Rep.* **7**, 41637; doi: 10.1038/srep41637 (2017).

**Publisher's note:** Springer Nature remains neutral with regard to jurisdictional claims in published maps and institutional affiliations.

## Supplementary Material

Supplementary Information

## Figures and Tables

**Table 1:** Demographics, risk factors, gait and balance function of the study sample.

Demographics	N = 678	%
Sex, male, N (%)	359	52.9
Age in years	72.5 (0.7)	
**Risk factors**	**N**	**%**
Smoking (current or ex), N (%)	359	52.9
High Blood Pressure, N (%)	332	48.9
Diabetes, N (%)	70	10.3
High Cholesterol, N (%)	284	41.9
Gait Speed and Balance function		
Gait speed (sec)	4.3 (1.2)	
Chair-Stands (sec)	13.7 (4.4)	
Standing balance (0–4)	3.8 (0.5)	

Sample characteristics are presented as mean and standard deviation (SD in brackets) if not otherwise specified.

N = number of subjects.

**Table 2:** MRI characteristics, prevalence of single SVD markers, SVD subscores and SVD specification of the study sample.

MRI characteristics		
Normalized Brain Volume (nIcV in %)	69 (1)	
WMH volume (cm^3^)	12.19 (12.17)	
**WMH score deep WMH**	**N**	%
0	104	15.3
1	430	63.4
2	124	18.3
3	20	2.9
WMH score perivent WMH		
0	21	3.1
1	436	64.3
2	178	26.3
3	43	6.3
CMB		
0	599	88.3
1–3	70	10.3
>3	9	1.4
Lacune		
0	639	94.2
1	28	4.1
≥2	11	1.6
PVS in BG		
0	2	0.3
1	402	59.3
2	249	36.7
3	24	3.5
4	1	0.1
**Score 1 on SVD scale**	**N**	**%**
**SVD WMH** (WMH score 2 or 3 in deep WMH &/or WMH score 3 in perivent WMH)	152	22.4
**SVD CMB** (1 or more CMB)	79	11.7
**SVD lacune** (1 or more lacunes)	39	5.8
**SVD PVS** (grade 2–4 in BG)	274	40.4

Sample characteristics are presented as mean and standard deviation (SD in brackets) if not otherwise specified.

BG = basal ganglia; Normalized Brain Volume (nIcV) = brain volume normalized by intracranial volume; lacune = lacune of presumed vascular origin, CMB = cerebral microbleeds, N = number of subjects, perivent = periventricular; PVS = perivascular spaces, WMH = white matter hyperintensities.

**Table 3:** Association of SVD related abnormalities with gait, chair-stands and balance test.

MRI parameters	R^2^	*βj*	p
Gait speed			
WMH volume cm^3^	2.3	0.16	0.001*
NBV	0.6	−0.09	0.036
WMH score	1.3	0.12	0.001*
CMB sum	ns		0.344
Lac sum	ns		0.574
PVS	ns		0.069
global SVD score	1.4	0.13	0.000*
Chair-Stands			
WMH volume cm^3^	ns		0.084
NBV	ns		0.687
WMH score	0.7	0.09	0.027
CMB sum	ns		0.207
Lac sum	ns		0.562
PVS	ns		0.279
global SVD score	0.6	0.09	0.028
Balance			
WMH volume cm^3^	ns		0.999
NBV	ns		0.505
WMH score	ns		0.983
CMB sum	ns		0.999
Lac sum	ns		0.997
PVS	ns		0.999
global SVD score	ns		0.877

Adjusted R^2^ (explanation of variance) and standardized beta-values (*βj*) are presented for significant findings only. Results marked with an asterisk (*) indicate significance with application of a Bonferroni-adjusted level of significance (0.007).

Lac = Lacunes of presumed vascular origin, CMB = cerebral microbleeds, NBV = brain volume normalized by intracranial volume in %, PVS = perivascular spaces in basal ganglia, WMH = white matter hyperintensities.

**Table 4:** Association of SVD related abnormalities with gait and balance function in addition to sex, age, risk factors andnormalized brain volume.

Demographics		Risk Factors		MRI parameter	
R^2^	*βj*	R^2^ (Δ R^2^)	*βj*	R^2^ (Δ R^2^)	*βj*
Gait					
5.4	age 0.15sex 0.15	6.8 (1.4)	HBP 0.12	9.7 (2.9)	NBV*−0.12**WMH vol cm**^3^*0.14
6.0	age 0.17sex 0.16	6.8 (0.8)	HBP 0.09	8.3 (1.5)	NBV*−0.11WMH score*0.10
6.0	age 0.16sex 0.16	6.8 (0.8)	HBP 0.09	8.8 (2.0)	NBV*−0.11global SVD score*0.12
Chair-Stand					
0.1	sex 0.09	ns		nsns	**NBVWMH vol cm**^**3**^
0.1	sex 0.08	ns		nsns	**NBVWMH score**
0.1	sex 0.08	ns		nsns	**NBVglobal SVD score**
Balance					
ns		ns		nsns	**NBVWMH vol cm**^**3**^
ns		ns		nsns	**NBVWMH score**
ns		ns		nsns	**NBVglobal SVD score**

Adjusted R^2^ (explanation of variance) and standardized beta-values (*βj*) are presented for significant findings only. Incremental explanation of variance is shown as delta (∆) of adjusted R^2^. Slight variations of the standardized beta-values are due to deviations of sample size. Results marked with an asterisk (*) indicate significance with application of an Bonferroni-adjusted level of significance (0.017).

HBP = high blood pressure; NBV = normalized brain volume.
