# Supplementary material for: Impact of small vessel disease in the brain on gait and balance
Source: Sci Rep. 2017 Jan 30;7:41637. doi: 10.1038/srep41637 (PMC5278543; doi:10.1038/srep41637)
Supplement: Supplementary Information [file srep41637-s1.pdf]

SUPPLEMENTARY INFORMATION

Impact of small vessel disease in the brain on gait and balance

Daniela Pinter [1], Stuart J. Ritchie [2,3], Fergus Doubal [2,5], Thomas Gattringer [1], Zoe Morris [4,5], Mark E. Bastin [4,5], Maria del C. Valdés Hernández [4,5], Natalie A. Royle [4,5], Janie Corley [b,c], Susana Muñoz Maniega [4,5], Alison Pattie [2,3], David A. Dickie [4,5], Julie Staals [6], Alan J. Gow [2,7], John M. Starr [2,8], Ian J. Deary [2,3], Christian Enzinger [1,9], Franz Fazekas [1], Joanna Wardlaw [2,4,5]*

[1] Department of Neurology, Medical University of Graz, Graz, 8036, Austria
[2] Centre for Cognitive Ageing and Cognitive Epidemiology, University of Edinburgh, Edinburgh, EH8 9JZ, UK
[3] Department of Psychology, University of Edinburgh, Edinburgh, EH8 9JZ, UK
[4] Brain Research Imaging Centre, University of Edinburgh, Edinburgh, EH4 2XU, UK
[5] Centre for Clinical Brain Sciences, University of Edinburgh, Edinburgh, EH4 2XU, UK
[6] Department of Neurology, Maastricht University Medical Centre, Maastricht, 6202 AZ, the Netherlands
[7] Department of Psychology, Heriot-Watt University, Edinburgh, EH14 4AS, UK
[8] Alzheimer Scotland Dementia Research Centre, Department of Psychology, University of Edinburgh, Edinburgh, EH8 9JZ, UK
[9] Division of Neuroradiology, Vascular and Interventional Neuroradiology, Department of Radiology, Medical University of Graz, Graz, 8036, Austria

* joanna.wardlaw@ed.ac.uk

Supplemental Methods

Assessment of gait and balance function

Gait speed was assessed by the six-meter walk test, a common assessment used in research studies to access physical activity[1]. The participants were asked to walk as quickly as possible with the use of a cane or walker if appropriate. Gait speed was measured in seconds. Furthermore, two subtests (chair- stands and standing balance) of the Short Physical Performance Battery were applied[2]. The chair-stands test assesses how long (seconds) it takes the participant to stand up and sit down as quickly as possible five times without stopping. Subjects had to keep their arms folded across their chest as demonstrated by the instructor; the number of stands was counted out loud. Tests of standing balance included side-by-side, semi-tandem, and tandem stands. All stands were demonstrated to the participant and they were asked to try to hold each position until the instructor told them to stop. For the side-by-side stand subjects were asked to stand with feet together, side-by-side for 10 seconds. For the semi-tandem stand subjects were asked to stand with the side of the heel of one foot touching the big toe of the other foot for about 10 seconds. For the tandem stand subjects were asked to stand with the heel of one foot in front of and touching the toes of the other foot for about 10 seconds. For the latter two, subjects could put either foot in front, whichever would feel more comfortable. For each position participants were instructed to move their arms, bend their knees or move their body to maintain balance, but not to move their feet. The standing balance test was scored from one to four points. One point was given if the side-by-side stand was held for ten seconds, a second if the semi-tandem stand was held for 10 seconds. One

point was given if the tandem stand was held between 3 and 9 seconds and a second if the tandem stand was held for 10 seconds.

Statistical Analysis

We checked for fulfilment of different assumptions for the regression analyses (e.g. linearity, homoscedasticity, auto-correlation (Durban-Watson-test), multicollinearity (Tolerance and Variance Inflation Factor)). Standardized beta-values (βj), adjusted R^2 (explanation of variance) and delta (Δ) adjusted R^2 (displaying incremental explanation of variance) in percent are presented for each model in the results section.

Supplemental Results

Supplemental Table S1: Correlations between gait speed and balance function with demographics and risk factors

	Gait Speed		Chair Stands		Standing Balance	
	r	***p***	***r***	***p***	***r***	***p***
**Age**	**0.154**	0.000	**0.070**	0.047	-0.051	0.135
**Sex**	**0.188**	0.000	0.065	0.063	**-0.114**	0.001
**Smoking**	0.066	0.054	**0.079**	0.025	0.021	0.543
**HBP**	**0.140**	0.000	0.065	0.063	**-0.090**	0.008
**Diabetes**	**0.159**	0.000	**0.090**	0.010	**-0.071**	0.039
**High Cholesterol**	0.042	0.215	-0.005	0.876	-0.065	0.059

HBP = High Blood Pressure.

Supplemental Table S2: Correlations between gait speed and balance function with single and combined SVD scores

	Gait Speed		Chair Stands		Standing Balance	
	r	***p***	***r***	***p***	***r***	***p***
**WMH vol cm^3**	**0.157**	0.000	**0.087**	0.027	**-0.085**	0.030
**NBV**	**-0.090**	0.036	-0.018	0.687	-0.020	0.635
**WMH score**	**0.149**	0.000	0.076	0.054	-0.053	0.168
**PVS**	**0.080**	0.037	0.034	0.392	-0.020	0.600
**CMB sum**	0.041	0.285	0.012	0.764	-0.056	0.150
**Lac sum**	0.053	0.752	0.120	0.481	0.110	0.504
**SVD global**	**0.147**	0.000	**0.087**	0.028	**-0.800**	0.038

Lac = Lacunes, CMB = cerebral microbleeds, NBV = normalized brain volume, PVS = perivascular spaces, WMH = white matter hyperintensities.

Supplemental References:

1. Tiedemann, A., Shimada, H., Sherrington, C., Murray, S. & Lord, S. The comparative ability of eight functional mobility tests for predicting falls in community-dwelling older people. *Age Ageing* **37,** 430–435 (2008).
2. Guralnik, J. M. *et al.* A Short Physical Performance Battery Assessing Lower Extremety Function: Association With Self-Reported Disability and Prediction of Mortality and Nursing Home Admission. *J. Gerontol.* **49,** 85–94 (1994).
